# Modelling the three-dimensional structure of the right-terminal domain of pospiviroids

**DOI:** 10.1038/s41598-017-00764-x

**Published:** 2017-04-06

**Authors:** Gerhard Steger

**Affiliations:** Institut für Physikalische Biologie, Heinrich-Heine-University Düsseldorf, 40225 Düsseldorf, Germany

## Abstract

Viroids, the smallest know plant pathogens, consist solely of a circular, single-stranded, non-coding RNA. Thus for all of their biological functions, like replication, processing, and transport, they have to present sequence or structural features to exploit host proteins. Viroid binding protein 1 (Virp1) is indispensable for replication of pospiviroids, the largest genus of viroids, in a host plant as well as in protoplasts. Virp1 is known to bind at two sites in the terminal right (TR) domain of pospiviroids; each site consists of a purine- (R-) and a pyrimidine- (Y-)rich motif that are partially base-paired to each other. Here we model the important structural features of the domain and show that it contains an internal loop of two Y · Y *cis* Watson-Crick/Watson-Crick (cWW) pairs, an asymmetric internal loop including a cWW and a *trans* Watson/Hoogsteen pair, and a thermodynamically quite stable hairpin loop with several stacking interactions. These features are discussed in connection to the known biological functions of the TR domain.

## Introduction

**Figure 1:**
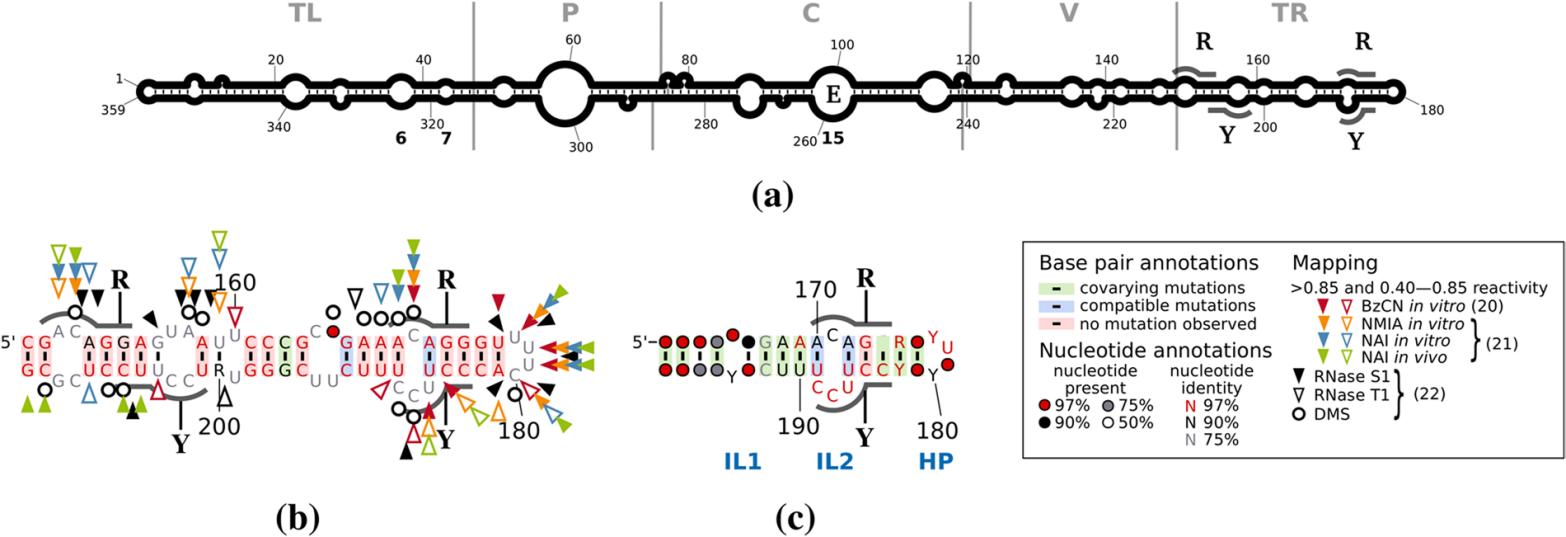
Secondary structure of pospiviroids. The consensus sequence and structure presented were predicted for alignments with MAFFT^66^, optimized in ConStruct^67^ at 37 °C, excluding lonely base pairs, and drawn with R2R^68^. (**a**) Structure of circular PSTVd. Borders of the five domains^74^ (TL, terminal left; P, pathogenicity-related; C, central; V, variable; TR, terminal right) are marked by gray lines. Loops 6, 7, and 15 (E) are marked. RY motifs critical for binding of Virp1^12^ are outlined and marked by R and Y. (**b**) TR domain based on an alignment of 234 full-length unique PSTVd sequences. High and low SHAPE reactivity is marked by filled and open triangles, respectively [red^62^; orange, blue and green^75^]; reactivity with RNase S1 (black triangle), RNase T1 (open triangle), and dimethyl sulfate (DMS, circle) is marked^76^. (**c**) Terminal right stem-loop based on an alignment (see Supplementary Fig. S2) of unique TR sequences of 95 pospiviroids (14 PSTVd, 25 CEVd, 21 columnea latent viroid (CLVd), 8 chrysanthemum stunt viroid (CSVd), 3 iresine viroid (IrVd), 3 Mexican papita viroid (MPVd), 10 pepper chat fruit viroid (PCFVd), 4 tomato apical stunt viroid (TASVd), 7 tomato chlorotic dwarf viroid (TCDVd)). The internal loops are marked by IL1 and IL2, respectively, and the hairpin loop by HP. The color code used in (b) and (c) for the annotation of nucleotides, base pairs and mapping is given in the right box.

Viroids, the smallest RNA pathogens known, consist of a circular, non-coding, single-stranded RNA (ssRNA) with a length of about 250–400 nucleotides depending on the viroid species^1, 2^. They are replicated in their plant hosts in an RNA-to-RNA rolling circle mechanism using either an asymmetric or a symmetric pathway^3^. In either pathway, replication includes a processing step of cleaving oligomeric replication intermediates to molecules of unit length. This step proceeds either by a viroid-internal ribozyme or by proteinaceous nucleases of the host. Based on this feature, viroids are classified into two families^4^: members of *Pospiviroidae* possess a thermodynamically stable rod-like secondary structure (Fig. 1a), replicate in the nucleus, and do not self-cleave; members of *Avsunviroidae* replicate in the chloroplast and self-cleave *via* a hammerhead ribozyme. The families are named after their respective type members potato spindle tuber viroid (PSTVd) and avocado sun blotch viroid. *Pospiviroidae* are divided into the genera *Pospi*-, *Hostu*-, *Cocad*-, *Apsca*-, and *Coleviroids*, based on sequence identity and biological properties^4^. All information for replication by cellular enzymes is included in structural features of the circular viroid RNA, which is abundant in the plant, and the less abundant oligomeric linear ssRNA replication intermediates of (+) and (−) polarity.

Only a few proteins or enzymes involved in the replication cycle of viroids are known. For example, members of *Pospiviroidae* and *Avsunviroidae* are transcribed by DNA-dependent RNA polymerase II and chloroplastic nuclear-encoded polymerase, respectively^5, 6^. Note that both enzymes are redirected to use ssRNA as template. Ligation of unit-length monomers to circles is supported by DNA ligase in case of PSTVd^7^ and by chloroplastic plant tRNA ligase for members of the *Avsunviroidae* family^8^, respectively.

Virp1 (Viroid binding protein 1) was isolated by screening a tomato cDNA expression library with PSTVd RNA^9^. The protein binds specifically (+) sense RNA of *Pospi*- and *Hostuviroid* members, but not of *Apscaviroid* and *Avsunviroidae* members^10^. Using electrophoretic mobility shift assays (EMSA) and the three-hybrid interaction system, the PSTVd region binding to the protein was determined: this is the terminal right (TR) domain of the rod like structure^11^ (Fig. 1a,b). Two binding sites, named RY motifs (Fig. 1), composed of the sequence elements 5′ ACAGG and 5′ CCUUCUC were found. In the RY motif close to the terminal right hairpin loop, the 3′ part of the purine-rich R sequence is paired to the 5′ part of the pyrimidine-rich Y sequence^12^; the same pairing for the inner RY motif is only present in suboptimal foldings. The binding affinity of Virp1 was about fivefold stronger to the terminal RY motif than to the inner motif. Furthermore, Virp1 was shown to be indispensable for replication of PSTVd and citrus exocortis viroid (CEVd), a further *Pospiviroid*, in *Nicotiana benthamiana* host plants as well as in protoplasts^13^. Similarly, the nuclear import of the satellite RNA of cucumber mosaic virus was shown to depend on Virp1^14^. Furthermore, the transcription factor TFIIIA binds to the TR in the region G_167_–U_176_^15^.

Virp1 (see Supplementary Fig. S1) is a member of the **b**romodomain(s) and **e**xtra**t**erminal domain (BET) protein family including a nuclear localization signal. BET proteins are known to be involved in chromatin biology and transcriptional regulation^16, 17^. The bromodomain recognizes acetyl-lysine residues in histones and other proteins^18, 19^. The ET domain consists of three regions; the conserved N-terminal ET (NET) region has an acidic patch that may interact with other proteins or nucleic acids^20^. The RNA binding domain of Virp1 has been localized to the C-terminal half of the protein^9, 12^.

The knowledge on the three-dimensional structure of viroids is sparse. At least for PSTVd the presence of a tertiary structure (in the sense of tRNA-L-conformation) was excluded with certainty^21^. The left terminal part of the TL domain was shown by nuclear magnetic resonance (NMR) spectroscopy to be rod-like but not bifurcated^22^; that is, this study supported secondary structure predictions but delivered no three-dimensional details. Loop 6 (Fig. 1a) has the sequence 5′ G_36_AC 3′/5′ C_322_GA 3′ flanked by a U:G wobble pair and a *cis* Watson-Crick/Watson-Crick (cWW) G:C pair; according to structural modelling, the loop motif is a set of three stacked non-WC pairs^23^. This motif mediates trafficking from palisade to spongy mesophyll in *N*. *benthamiana*. Loop 7 (Fig. 1a) is required to traffic from nonvascular into the vascular tissue phloem to initiate systemic infection. According to modelling, the loop nucleotides U_43_ · C_317_ are a water-inserted cWW base pair flanked by canonical cWW base pairs^24^. Loop 15 of PSTVd (Fig. 1a) is similar to loop E (sarcin/ricin motif) of eukaryotic 5 S rRNA^25–27^. A detailed analysis of this loop, mainly based on isostericity matrices, allowed Zhong *et al*.^28^ to design disruptive as well as compensatory mutations that are critical in rolling-circle replication^29–31^.

We created three-dimensional RNA models of the terminal right domain of several circular viroids, using a variety of recently published web services, programs and databases, for example FARFAR/Rosetta^32–34^, RNA Bricks^35^, FRAbase (RNA FRAgments search engine & dataBASE)^36^, based on sequence and two-dimensional structural similarity to known three-dimensional RNA structures. These programs were selected for use mainly based on their complementarity (assembly of 3-nt fragments plus refinement versus search for similar structural elements using sequence and structure patterns) from a palette of available tools^37–42^. The predicted models encompassed three loops: a symmetrical basepaired 2×2 loop, an asymmetrical internal loop, and a hairpin loop both stabilized by hydrogen bonding and stacking interactions. On the one hand this predicted structure should provide an extraordinary platform for protein and/or nucleic acid binding *in trans*, on the other this model should facilitate further experimental and structural studies of the TR interaction with Virp1 and TFIIIA.

## Results

### Secondary structure prediction of TR domain

**Figure 2:**
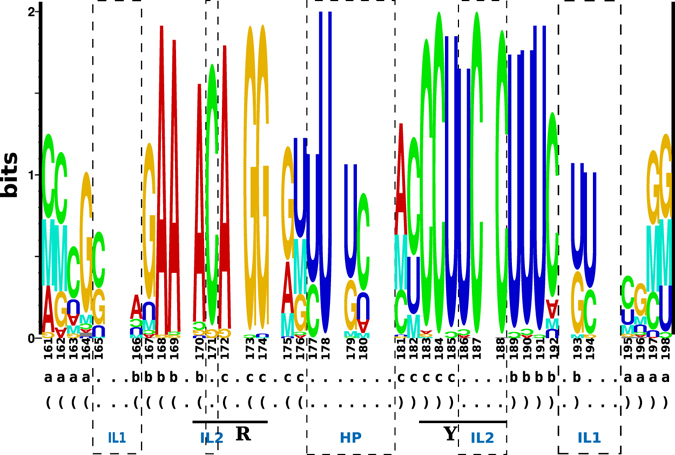
Structure logo of terminal right stem-loop. This logo^46^ is based on the same alignment (see Supplementary Fig. S2) of 95 pospiviroids as used for Fig. 1c. The height of nucleotide characters is proportional to the observed frequency of nucleotides in the alignment and the expected (a priori) frequency *p* = 0.25; nucleotide characters that appear less than expected are displayed up-side-down. The mutual information (marked by ‘M’) between base-paired positions, as indicated in the two bottom lines, is calculated as the relative entropy between the fractions of complementary bases and the number of basepairs one would expect by chance from the distribution of nucleotides at the involved positions. The nucleotide numbering is as in Fig. 1c; missing numbers denote that the respective column of the alignment contains mostly gaps. The RY motif critical for binding of Virp1 is outlined and marked by R and Y. The sequences of internal loops are marked by IL1 and IL2, respectively, and the hairpin loop sequence by HP.

To get an overview on available TR sequences from members of *Pospiviroid* and their secondary structures, we assembled alignments of full-length sequences for each species of *Pospiviroid* and extracted the TR domain. For example, all unique sequences of PSTVd were aligned and a consensus structure was predicted; Fig. 1b details the TR domain of the PSTVd consensus structure. The TR stem-loop consisted of six well-defined helices but the sequences of loops more distant from the hairpin loop were difficult to align, especially if sequences of further *Pospiviroid* members were included. This is of concern only to the Virp1 binding site of lower affinity (nts 149–153/202–206), while the binding site of higher affinity (nts 170–174/183–187) is located in the more easily alignable region of the terminal stem-loop (nts 161–198; Fig. 1c). We will concentrate in the following on this terminal stem-loop that consists of three helices separated by two internal loops (IL1 and IL2) and the hairpin loop (HP). The final alignment of terminal stem-loop sequences (see Supplementary Fig. S2), on which the consensus structure (Fig. 1c) and the structure logo (Fig. 2) are based, contained 95 different sequences of pospiviroids.

The nucleotides in the two helices close to the HP are well conserved in sequence (marked by b and c in Fig. 2) but show some covariation marked by green and blue background in Fig. 1c. The sequence of the terminal HP loop is 5′ UUUC in most pospiviroids, with the exception of CEVd and IrVd that have 5′ CUCGW (W is A or U). The sequence of IL1 is 5′ GA/GC in CEVd and 5′ CW/UU in PCFVd, PSTVd, and most CLVd. The sequence of IL2 is 5′ C/UCC, conserved in most pospiviroids. This degree of conservation in sequence as well as in secondary structure of the TR stem-loop gave some confidence as a basis for modelling.

### Results from Rosetta modelling

**Figure 3:**
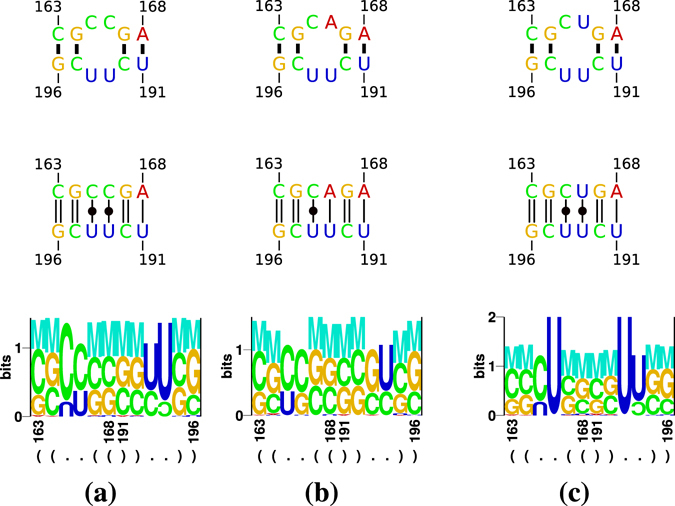
Results of Rosetta modelling of IL1 variants. Results of Rosetta modelling are shown for three typical loop variants: CC/UU (**a**), CA/UU (**b**), and CU/UU (**c**). Top: secondary structure of IL1 used as input to Rosetta modelling; that is, the input structure was ((..((+))..)) for all three sequences. Middle: interactions identified by rnaview in Rosetta’s top model; standard cWW base pairs are marked by double lines; all “loop” nucleotides are paired in cWW conformation; for the basepairing nomenclature of Leontis & Westhof^77^ see Supplementary Fig. S1. Bottom: Logos produced by RNAredesign for Rosetta’s top model.

**Figure 4:**
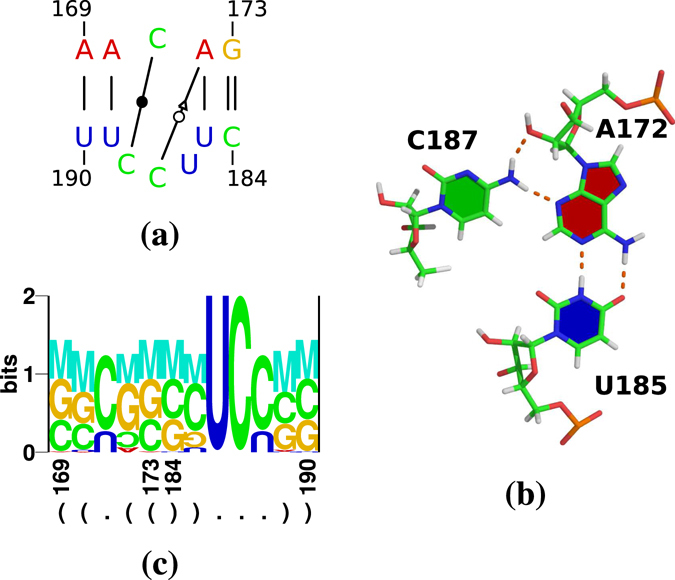
Results of Rosetta modelling of IL2 loop. (**a**) Interactions identified by rnaview in Rosetta’s top model. Loop nucleotides were highly connected by a complex network of base/base hydrogen bonds and stacking interactions. (**b**) Triple pair C_187_ · A_172_:U_185_ (cWW A_172_:U_185_, *trans* Watson/Sugar edge (tWS) C_187_ · A_172_). (**c**) Logo produced by RNAredesign for Rosetta’s top model.

**Figure 5:**
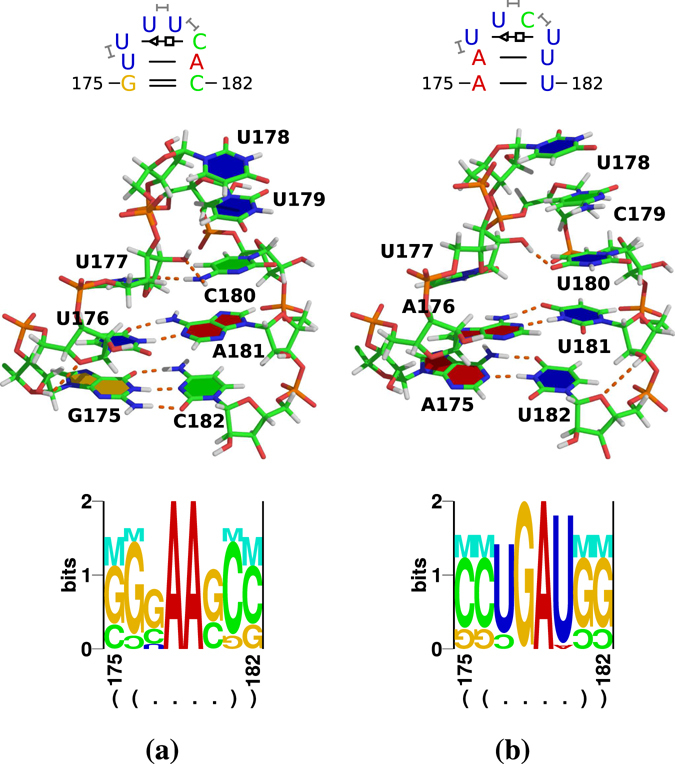
Results of Rosetta modelling of HP loop. Results for a typical loop of most pospiviroids is shown in (**a**); an exceptional loop of TCDVd is shown in (**b**). Top: Interactions identified by rnaview and x3dna-dssr. Middle: 3D view produced by PyMOL. Bottom: Logos produced by RNAredesign.

We predicted models of IL1 (Fig. 3), IL2 (Fig. 4), and HP (Fig. 5) within the TR stem-loop (Fig. 1c) with the Rosetta web server (ROSIE/FARFAR/RNA De Novo). Fragment assembly for the full TR stem-loop showed only a limited convergence; with shorter structural elements, like the single loops, 1,000 generated structures and 10,000 MC cycles were usually sufficient to generate reasonable best-scoring structures. As input to the web server, we used the respective parts of the consensus structure (in bracket-dot notation) but sequences from individual viroid variants (for examples see Fig. 3, top row).

#### Internal loop 1 (IL1)

The IL1 loop typical for PSTVd is a symmetric internal loop of 2×2 nucleotides (10× CC/UU; Fig. 1b; Fig. 3a, top row). Other loop nucleotides also occur frequently (Fig. 2): 15× CU/UU (Fig. 3c); 2× UU/UU; a 1×1 loop of C/U followed by an A:U pair (13×; Fig. 3b); a G:C pair followed by 1×1 loop of A/G is typical for CEVd (19×). The loop is closed by G:C cWW pairs but also other closing pairs appear quite often. The loop nucleotides form standard cWW pairs in all models of these sequence variants (Fig. 3, middle row). Thus other cWW pairs should be able to substitute: U · C, G · A, A · G, and A · A are isosterically close to C · U; G · U, A · C, and C · C are isosterically close to U · U^43, 44^. These isosteric pairs, however, do not occur in viroids. Only a restricted set of pair combinations are able to retain the same backbone geometry according to RNAredesign^45, 46^; this is obvious from the logos shown in Fig. 3 bottom. For example, CC/UU might be replaced by UU/CC or CU/UU by UU/UC. Thus, the viroid-specific combinations of nucleotides might be either essential for interaction with Virp1, or the reduced stability of IL1—in comparison to an IL1 composed of purines with their increased stacking—is critical.

The stability of such tandem “mismatches” is partially known^47, 48^: the tandem U · U stabilizes a helix when flanked by G · C pairs, as in case of viroids, but is less stable than symmetric tandem G · U or A · G pairs. The pyrimidine base-pairs led to a contraction of the sugar phosphate backbone of the opposing strands in comparison to standard cWW pairs; this might be of importance for protein binding.

#### Internal loop 2 (IL2)

Predicted IL2 loop nucleotides were highly connected by a complex network of base/base hydrogen bonds and stacking interactions (Fig. 4a). An example of these interactions is shown in Fig. 4b: C_187_ · A_172_:U_185_ form a triple pair consisting of a cWW A:U and a *trans* Watson/Sugar edge (tWS) C · A.

According to RNAredesign, U_186_ and C_187_ are critical for the backbone geometry of the loop (Fig. 4c). The cWW pair C_171_ · C_188_ is replaced by a U · U in 22% of structures, which is the only near-isosteric pair to C · C^44^.

#### Hairpin loop (HP)

The most common HP loop is UUUC closed by an U:A pair (Fig. 5a); the HP loop of CEVd and IrVd is CUCGW closed by a G:C pair (see Supplementary Fig. S2). The predicted HP loop structures were characterized by internal interactions of loop nucleotides. For the most common HP loop, Rosetta predicted a conformation with a *trans* Sugar/Hoogsteen (tSH) pair U_177_:C_180_ next to the loop-closing cWW U:A pair and stacking of the two last loop nucleotides (Fig. 5a). The exceptional HP loop of a TCDVd (Fig. 5b) was very similar with a tSH pair U_177_:C_180_ next to the loop-closing A:U pair and stacking of the two last loop nucleotides. For the HP loop of CEVd, Rosetta predicted a quite similar structure, despite the five loop nucleotides instead of the tetra-loops in most other cases: the fifth nucleotide of the loop was bulged out (see Supplementary Fig. S3b).

RNAredesign suggested quite different sequences for all loop models shown in Fig. 5 and S3: the optimized sequences contained more purines than the natural sequences, which might point on the one hand to increased thermodynamic stability of the optimized sequences due to increased stacking interactions, and on the other to functional constraints of the natural sequences. Such constraints might be an adaptation to stability requirements and restrictions induced by binding to other molecules.

### Structural similarities in RNABricks and FRAbase

The Rosetta models, as depicted above, are based on assembly of short fragments from known structures. These fragments might include nucleotides that interact with additional ligands like a protein or another RNA. That is, “unusual” conformations of nucleotides in the final models might be due to absent ligands. In contrast, if a larger fragment of the target is found in a known (template) structure including ligands, one might get insight on the basis of such “unusual” conformations and requirements for interactions. So we searched for structural elements similar to the terminal stem-loop in RNABricks^35^ and FRAbase^36^.

#### IL1

An internal loop with a 5′ CU/5′ UU motif, frequent in pospiviroids, is described as a non-canonical tandem base-pair (i. e. two cWW pairs) in the 3′-UTR of poliovirus-like enteroviruses^49^. Remarkably, this loop is conserved but replacement of the C · U and U · U pairs by a C · G and U · A pairs, respectively, had no effect on virus viability and growth in cell culture. This is in contrast with viroids: replacement of IL1 by two A · U pairs diminished replication efficiency and abolished systemic infection^50^ (see below and Supplementary Table S4).

The intron 1 of human cellular nucleic acid-binding protein (also named zinc finger protein 9) contains a (CCUG)_*n*_ repeat; expansion of this repeat causes myotonic dystrophy type 2^51^. The repeat folds into a stem-loop with 5′ CU/5′ UC tandem cWW pairs flanked by GC pairs that binds and inactivates a splicing regulator^52^. This symmetric tandem mismatch, however, occurs only in CLVd JF446928.

#### IL2

**Figure 6:**
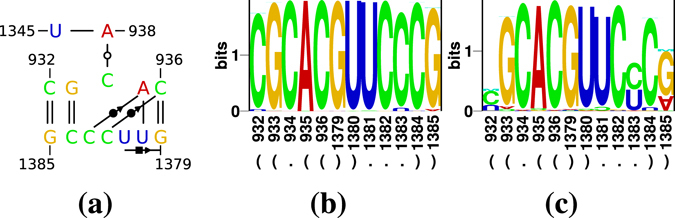
A motif from *Thermus thermophilus* 16S ribosomal RNA. (**a**) Nucleotides 932–936 and 1379–1385 are similar in sequence to IL2 of PSTVd; interactions are extracted by x3dna-dssr from PDB 1FJG^78^. (**b**) A logo of the motif (**a**) from an alignment of 23,500 bacterial 16S ribosomal RNAs^54^. (**c**) A logo of the motif (**a**) for 1850 sequences from the same alignment as used in (**b**), but sequences identical to the consensus 5′ CGCAC/GUUCCCG 3′ were removed. Columns with less then 1% of nucleotides and sequences not fully spanning the motif or containing nucleotides other then [AUCG] were removed from the alignments used for the logos.

RNA Bricks found an internal loop with some similarity to IL2 in PDB structures of 16S ribosomal RNA; for an example see Fig. 6a. The internal loop has the sequence C_934_/_1381_UCC_1383_ with a 3′ closing A:U pair as in PSTVd (Fig. 4) but with a 5′ G:C pair. C_934_ is bulged out from the motif and makes a tWW pair with A_938_:U_1345_ (see motif IL_40845.1 in the RNA 3D Motif Atlas)^53^. C_1382_ and C_1383_ pair with C_936_ and A_935_ in cWS conformation, respectively. U_1381_ pairs with G_1379_ in cHS conformation as annotated by x3dna-dssr; rnaview strangely determines this interaction as a cSW pair. A protein contact seems not relevant for this motif. The sequence of this loop is highly conserved according to an alignment of bacterial 16S ribosomal RNAs^54^; a corresponding logo is shown in Fig. 6b. The most variant loop nucleotide obviously is C_1383_ (Fig. 6b and c) that is replaced by an U in ~3% of sequences; this replacement did not occur in IL2.

#### HP

**Figure 7:**
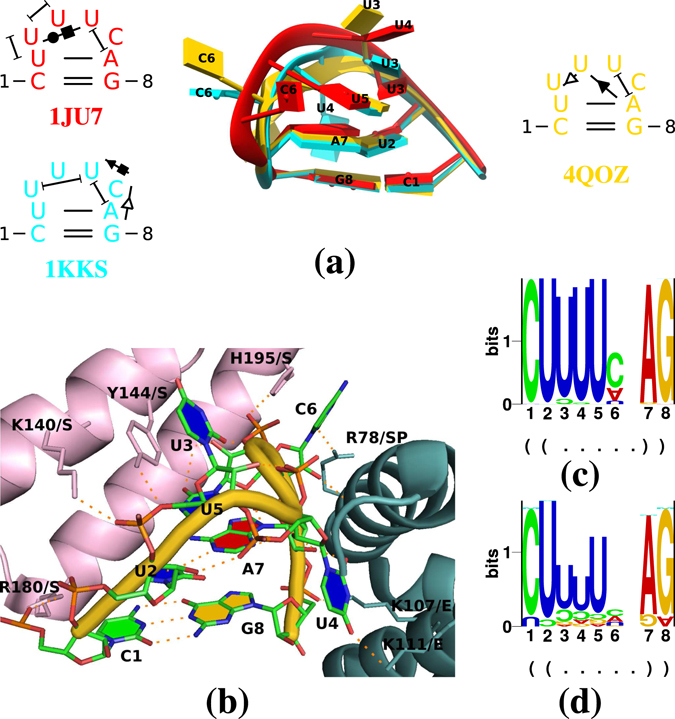
Histone mRNA stem-loop. This hairpin loop including the closing base pair is identical in sequence to PSTVd’s HP (Fig. 5a). (**a**) Comparison of hairpin loops of PDB entries 1JU7 (red), 1KKS (cyan), and 4QOZ (gold); model 5 of 1JU7 and model 8 of 1KKS are shown, which are annotated as best structures in their respective PDB files. Views from different angles are shown in Supplementary Fig. S4. (**b**) Hairpin loop (cartoon in gold) from 4QOZ, bound by Exo (cartoon in lightpink) and SLBP (cartoon in darkgreen); a few amino acid side chains, which interact with RNA backbone and bases, are labelled. (**c**) Logo of 11,099 histone mRNA loops from Rfam-11^79^ (ID: Histone3); three sequences have an additional nucleotide after position 6. (**d**) Logo of 36 non-redundant histone mRNA loops from Rfam-11.

Hairpin loops with the four nucleotides and closing base pair common in PSTVd’s HP (Fig. 5a) are present in the histone mRNA stem-loop (PDB entries 1JU7^55^ and 1KKS^56^), which binds to the stem-loop binding protein (SLBP) and the histone mRNA 3′-exonuclease 1 (Exo) for 3′ end processing. These hairpin structures—without corresponding proteins—were determined by NMR spectroscopy. An overlay of both hairpin loops is shown in Fig. 7a; for this overlay, the terminal C_1_:G_8_ pairs were matched in chimera. Note the stackings of U_2_, U_3_ and U_4_, and of U_5_ on A_7_ in 1JU7 (red), while U_3_, U_5_ and A_7_ stack in 1KKS (cyan). In 1JU7 the C_6_ is bulged out to the major groove, whereas in 1KKS the C_6_ is bulged out to the minor groove. According to FRAbase, further PDB entries (1ZBH, Cheng & Patel, unpublished; 4L8R and 4QOZ)^57^ are similar to HP; each of these contains two of the histone stem-loops bound by protein (for example see Fig. 7b). These loop structures are very close, but not identical to 1JU7 and 1KKS, respectively, due to the interactions with protein, like stacking of Tyr_144_(Exo)/U_3_ and His_195_(Exo)/C_6_ and numerous hydrogen bondings between protein side chains and RNA backbone and bases. The sequence of the histone mRNA stem-loop is highly conserved (Fig. 7c,d), which might be forced by interactions with the proteins. The most variable position is C_6_ (24% A, 68% C, 1% G, 7% U) as in HP (C_189_; Fig. 2). The differences between the sequence logos of the histone mRNA stem-loop and HP might be due to additional requirements of HP like stability in the (−) strand, but might also point to a clear biological difference (see Discussion).

**Figure 8:**
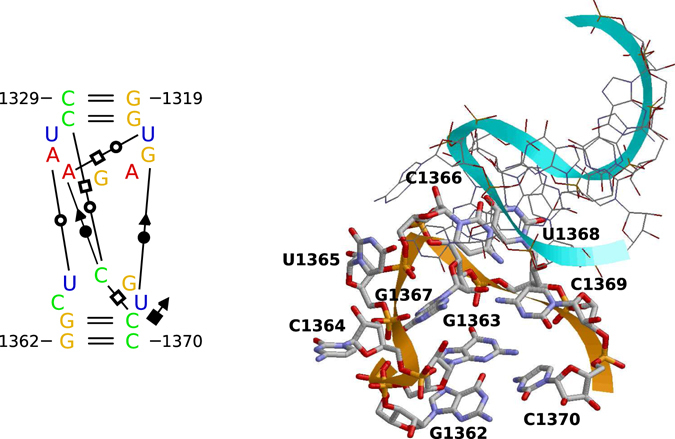
View on kissing-loop interaction. The nucleotides 1319–1329 and 1362–1370 from PDB entry 3J3W are visualized by rnaview (left) and by RasMol (right). The hairpin loop 1363–1369 is identical in sequence to the standard hairpin loop of CEVd.

We further searched for HP-loop variants from other viroids. The PDB entry 4V7E^58^, a model of the *Triticum aestivum* 80S ribosome based on cryo-electron microscopy with resolution of <5 Å, contains in the 28S rRNA a hairpin loop with sequence _767_UUCU_770_ closed by a C:G pair instead of the A:U pair in a TCDVd (ID GQ169709). The first and last loop nucleotide stabilize the loop by a cWW interaction, and U_769_ and U_770_ stack on each other (see Supplementary Fig. S5); both interactions might be independent from the loop-closing pair. The PDB entries 3J3V and 3J3W^59^, which contain structures of the *Bacillus subtilis* 50S ribosome subunit based on cryo-electron microscopy and usage of ribosome structures of *Escherichia coli* and *Thermus thermophilus* as templates, show in the 23S rRNA a hairpin loop, similar to that of CEVd (see Supplementary Fig. S3b), that is involved in a kissing loop interaction (Fig. 8). Most interactions in the rRNA loop are due to the kissing interactions; however, the last loop nucleotide, in the rRNA as well as in the predicted CEVd loop, makes a cSH interaction with the 3′ nucleotide of the loop-closing basepair.

### Synthetic mutants in the TR domain

#### A synthetic mutant of HP and its progeny

Hammond^60^ described a mutant PSTVd construct R+ (with the replacement UU_177_ → GAA in HP; see Supplementary Table S2) that was not infectious as transcribed RNA in contrast to the wild-type (WT) variant. After *Agrobacterium tumefaciens*-mediated inoculation of tomato plants with a corresponding plasmid containing a R+ cDNA insertion in (+) orientation, viroid progeny with secondary mutations appeared in galls and roots, and in 10% of infected plants also in leaves. The only variant (1 in Supplementary Table S2) appearing in leaves had, in comparison to WT, an inversion of the loop closing pair from U_176_:A_181_ in the WT to A:U, similar to the already mentioned TCDVd variant (Fig. 5b), and an additional U in the HP loop. The original variant R+, which was never recovered, and a further evolved variant (2 in see Supplementary Table S2) might be less viable due to a possible structural rearrangement influencing IL2 and the RY motif and thus did not point to critical loop nucleotides. The loop-closing pair and the loop size, however, seemed uncritical for replication and trafficking.

#### Binding affinity and infectivity of IL2 mutants

Gozmanova *et al*.^12^ analyzed the binding of a Virp1 fragment to mutants of stem-loops, which were similar to the hairpin depicted in Fig. 1b. That is, their TR fragment contained both RY motifs, but only the terminal motif (as in Fig. 1c) was mutated. Infectivity of longer-than-unit-length viroid transcripts and relative binding affinity of stem-loops are given in Supplementary Table S3. From these data and further biophysical experiments, the authors concluded that the binding to the terminal RY motif was about fivefold stronger than to the internal motif.

Rosetta modelling of the synthetic IL2 mutants, which include single nucleotide replacements or a reverse complement of the loop sequence, predicted that these mutants possess at least one non-WC interaction different from but also additional to WT. Both modified interaction types might lead to an increased stability, rigidity and/or bending of the mutant loops, explaining the reduced binding of mutants to Virp1.

#### Inhibition of replication and trafficking by mutations in the TR domain

Zhong *et al*.^50^ analyzed PSTVd loop motifs with respect to replication in protoplasts and to (replication plus) trafficking in plants. A replacement of the cWW pairs of IL1 by standard A · U WC pairs reduced replication efficiency to 29% of WT and abolished systemic infection. This experimental result fits to the findings from Rosetta modelling: either the lower thermodynamic stability of the WT loop is critical for replication competence, or the cWW loop pairs—in comparison to a loop without pairs—“protect” against endoribonucleases, or the backbone contraction in the WT loop is critical for protein binding. A replacement of the IL2 loop by three standard WC pairs reduced replication efficiency to 46% of WT and abolished systemic infection. A mutation of UU_176_ → AA, which opens at least the HP-closing U · A pair, reduced replication efficiency to 53% of WT and abolished systemic infection. An additional mutation, which occurred in one plant, restored the loop-closing pair by an A · U pair that allowed this PSTVd mutant for systemic infection. This analysis points to a lower importance of loops in the TR domain for replication than loops in the TL and central domain but shows that these are most critical for trafficking^50^.

## Discussion

In the following we will discuss selected features from the predictions and compare several results from Rosetta modelling with findings from the structure databases.

### Relevance of the two binding sites in TR

Figure 1b only shows the terminal Virp1 binding site with pairing of the R to Y motif, whereas the second, internal binding site is masked by different pairings. A structure with both Virp1 binding sites^12^, is inferior to the one shown here by about 10 kJ/mol (mean ΔΔ*G*_37°C_ of 234 PSTVd variants) or a ratio of both structure ensembles (with two versus one perfect binding site) of about 50. This fits roughly to the different binding of Virp1 to both sites^12^. However, only five of the 234 PSTVd variants miss the internal RY motif (ACs HQ452399–401, HQ452411, HQ452412)^61^, which points to the *in vivo* importance of a twofold RY motif.

### IL1 is closed by two cWW pairs

The majority of IL1 is a 2 × 2 C · U forming cWW pairs; alternatives are a single cWW plus a standard WC pair (for example 5′ CA/UU 3′, 5′ GA/GC 3′). These interactions fit to low BzCN reactivity of IL1^62^. A further “loop” stabilization by two A · U pairs with standard A-RNA geometry is prohibitive for viroid replication and abolishes trafficking^50^.

### Base-pairing in the asymmetric IL2 loop

According to the Rosetta model, only U_186_ is bulged out while the three other nucleotides are paired. In contrast, the C_171_ is bulged out in the 16S rRNA loop and makes contact to a distant base pair. In both cases, the logos for IL2 (Fig. 4c) and for the rRNA loop (Fig. 6b and c) do not point to compatible mutations of each motif. This fits to the higher degree of sequence conservation of IL2 than of IL1.

### The HP structure allows for interaction despite its stability

According to DeJong *et al*.^55^, the “stacking of loop nucleotides [in the histone mRNA stem-loop that is quite similar to HP] has the net effect of extending the helix by an additional base pair step”, which accounts for the relatively high thermodynamic stability of HP. However, the mRNA hairpin still allows for interaction with an RNA binding protein (Fig. 7) or for a kissing-loop interaction (Fig. 8).

In our opinion, however, the similarity between HP and histone mRNA stem-loop should not be taken as a hint towards biologically relevant interactions of viroid RNA with SLBP and Exo: (i) sequences similar to SLBP are not found by BLAST in higher plants; (ii) sequences similar to Exo are present in plant genomes, but these miss the SAP domain responsible for RNA binding; and (iii) histone 3′ end processing is believed to be restricted to metazoa and green algae^63, 64^. Thus the detected similarity might be a pure consequence of the recurrence of evolutionary unrelated RNA motifs^65^.

This work was inspired by the numerous tools for 3D RNA structure prediction that all promise to be manageable by non-experts. Indeed, our results point to an overall TR structure with a nice combination of rigidity and flexibility allowing the TR for binding of proteins. We have, however, not found a reason for the proposed TFIIIA binding to the TR region. Taken together, the predicted structure is encouraging for further exploration by mutational analysis and experimental structure determination.

## Methods

### Viroid sequences and consensus structures

For each of the 10 species of genus *Pospiviroid* ^4^ a single sequence was used to search with nucleotide BLAST for somewhat similar sequences in GenBank. For sequences from each species, a preliminary alignment was produced with MAFFT/G-INS-i^66^. According to these alignments, partial sequences were removed and the remaining sequences were adjusted for same strand orientation and similar start/end points. After a second alignment, redundant sequences were removed; that is, sequences from each genus had to differ from each other by at least one mutation. The final alignments were optimized in ConStruct^67^ at 37 °C, excluding lonely base pairs, and drawn with R2R^68^. An example is the consensus structure of PSTVd (Fig. 1a) which was based on 234 unique sequences. From the PSTVd alignment, the TR domain was cut out, redundant sequences were removed, aligned with MAFFT/X-INS-i^69^, and optimized in ConStruct (Fig. 1b). From all individual alignments, the range of the terminal stem-loop of three helices was cut out, pooled, redundant sequences were removed, and aligned with MAFFT/X-INS-i. This alignment is shown in Supplementary Fig. S2, the corresponding R2R drawing in Fig. 1c.

### Rosetta modelling

We used the Rosetta web server (ROSIE (Rosetta Online Server that Includes Everyone)/FARFAR (Fragment Assembly of RNA with Full Atom Refinement)/RNA De Novo) to predict 3D structures^33^. FARFAR^34^ assembles RNA fragments with length ≤3 nucleotides known from x-ray structures; these fragment sequences match partial sequences of the target RNA. The assembly is a Monte Carlo (MC) process guided by a knowledge-based energy function. Resulting structure models are then refined in an all-atom potential that is thought to discriminate native-like from non-native conformations. We used the following options: full run; variation of bond lengths and angles; optimization after fragment assembly; allowance for bulges; usage of updated (2012) force field; 1,000 structures; 10,000 MC cycles. From the predicted 3D structure(s), we extracted information on types of base pairs and further interactions by rnaview^70^ and x3dna-dssr^71^.

RNAredesign^45^ samples the possible sequence space that stabilizes a given 3D structure with a fixed structure backbone.

### Database searches

The databases RNABricks^35^ and FRAbase^36^ store short RNA motifs extracted from experimentally determined RNA 3D structures. Both databases allow a user to search for sequences similar to a query. Additionally, RNABricks includes a 3D search engine; as input we used 3D models predicted by ROSIE.

### Further software

chimera v1.10.1^72^, PyMOL (https://www.pymol.org/) with nuccyl (http://www.biosci.ki.se/groups/ljo/software/nuccyl.html), and RasMol v2.7.5^73^ were used to visualize 3D structures.

## Electronic supplementary material

Supplementary information
