# Supplementary material for: Modelling the three-dimensional structure of the right-terminal domain of pospiviroids
Source: Sci Rep. 2017 Apr 6;7:711. doi: 10.1038/s41598-017-00764-x (PMC5429643; doi:10.1038/s41598-017-00764-x)
Supplement: Supplementary file 1 — Supplementary information [file 41598_2017_764_MOESM1_ESM.pdf]

Supplementary information for

"Modeling the three-dimensional structure of the right-terminal domain of pospiviroids"

Gerhard Steger [1]

[1] Institut für Physikalische Biologie, Heinrich-Heine-University Düsseldorf, 40225 Düsseldorf, Germany
* Email: steger@biophys.uni-duesseldorf.de

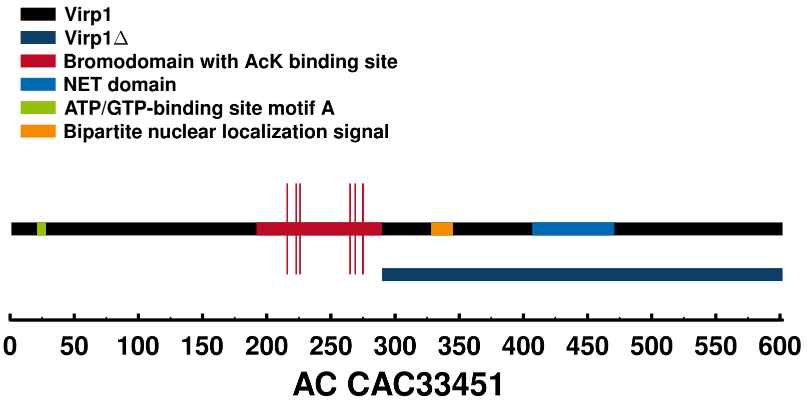

Figure S1. Structure of Virp1. Plant BET (bromodomain(s) and extraterminal domain) proteins typically have only one bromodomain (marked in red; amino acids 192–290; Prosite entry PS50014), whereas they have mostly two bromodomains in animals; six amino acids (marked by red lines; aa 216, 223, 226, 265, 269, and 275) are responsible for the acetyl-lysine recognition of histones and other proteins.[1] The structure of the extraterminal domain (lightblue; aa 407–471; PS51525) has an acidic patch that may interact with other proteins or nucleic acids.[2] Furthermore, SCANPROSITE[3] predicts an ATP/GTP-binding site (green; aa 21–28; PS00017) and a bipartite nuclear localization signal (orange; aa 328–344; PS50079). Virp1 Δ (aa 290–602) is sufficient for binding to PSTVd[4].

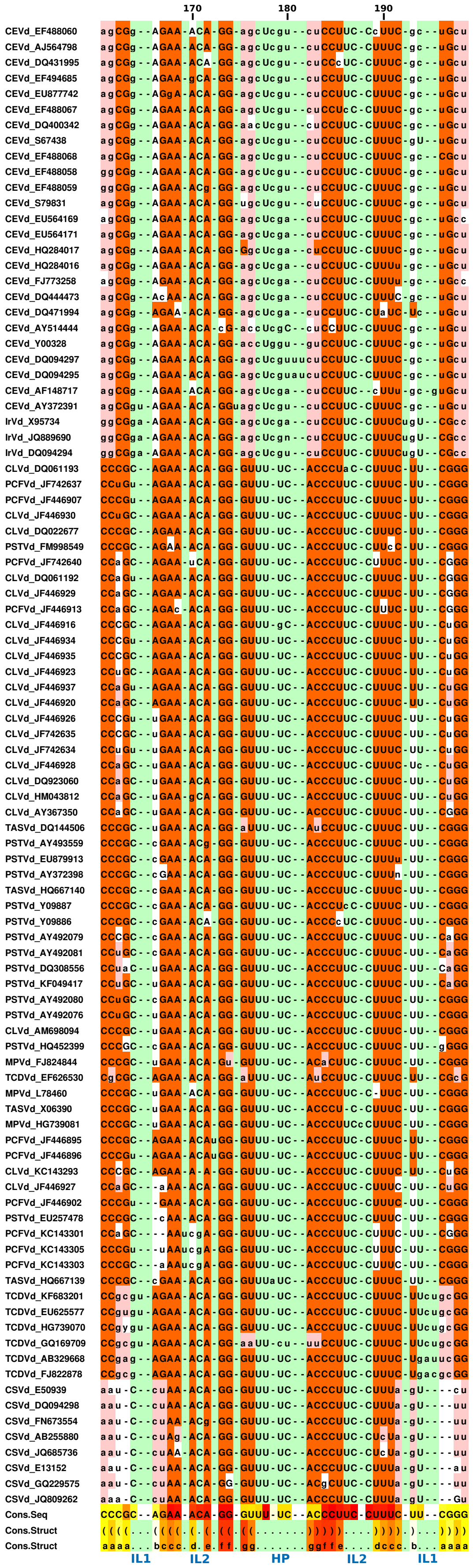

Figure S2. Structural alignment of terminal right hairpin. Left: Sequence names (viroid abbreviation + GenBank UID). From top to bottom: sequences [with background colors green for loops, red for consensus base pairs, pink for consensus base pair changes (covarying pairs), and white for non-base pairs in paired regions], the consensus sequence, and the consensus structure in bracket-dot notation and character-encoded (both with background colors from white to red proportional to sequence conservation resp. pairing probability). At the bottom are named the respective structural regions, for which ROSETTA modelling was done: IL1, internal loop 1; IL2, internal loop 2; HP, hairpin loop.

Table S1. Leontis & Westhof nomenclature[5]

cis	Watson-Crick/Watson-Crick	cWW	–●–
trans	Watson-Crick/Watson-Crick	tWW	–○–
cis	Watson-Crick/Hoogsteen	cWH	●–■
trans	Watson-Crick/Hoogsteen	tWH	○–□
cis	Hoogsteen/Watson-Crick	cHW	■–●
trans	Hoogsteen/Watson-Crick	tHW	□–○
cis	Watson-Crick/sugar	cWS	●–▶
trans	Watson-Crick/sugar	tWS	○–▷
cis	sugar/Watson-Crick	cSW	◀–●
trans	sugar/Watson-Crick	tSW	◁–○
cis	Hoogsteen/Hoogsteen	cHH	–■–
trans	Hoogsteen/Hoogsteen	tHH	–□–
cis	Hoogsteen/sugar	cHS	■–▶
trans	Hoogsteen/sugar	tHS	□–▷
cis	sugar/Hoogsteen	cSH	◀–■
trans	sugar/Hoogsteen	tSH	◁–□
cis	sugar/sugar	cSs	–▶–
trans	sugar/sugar	tSs	–▷–
cis	sugar/sugar	csS	–◀–
trans	sugar/sugar	tsS	–◁–
		GC	=
		AU	—
stacking			———

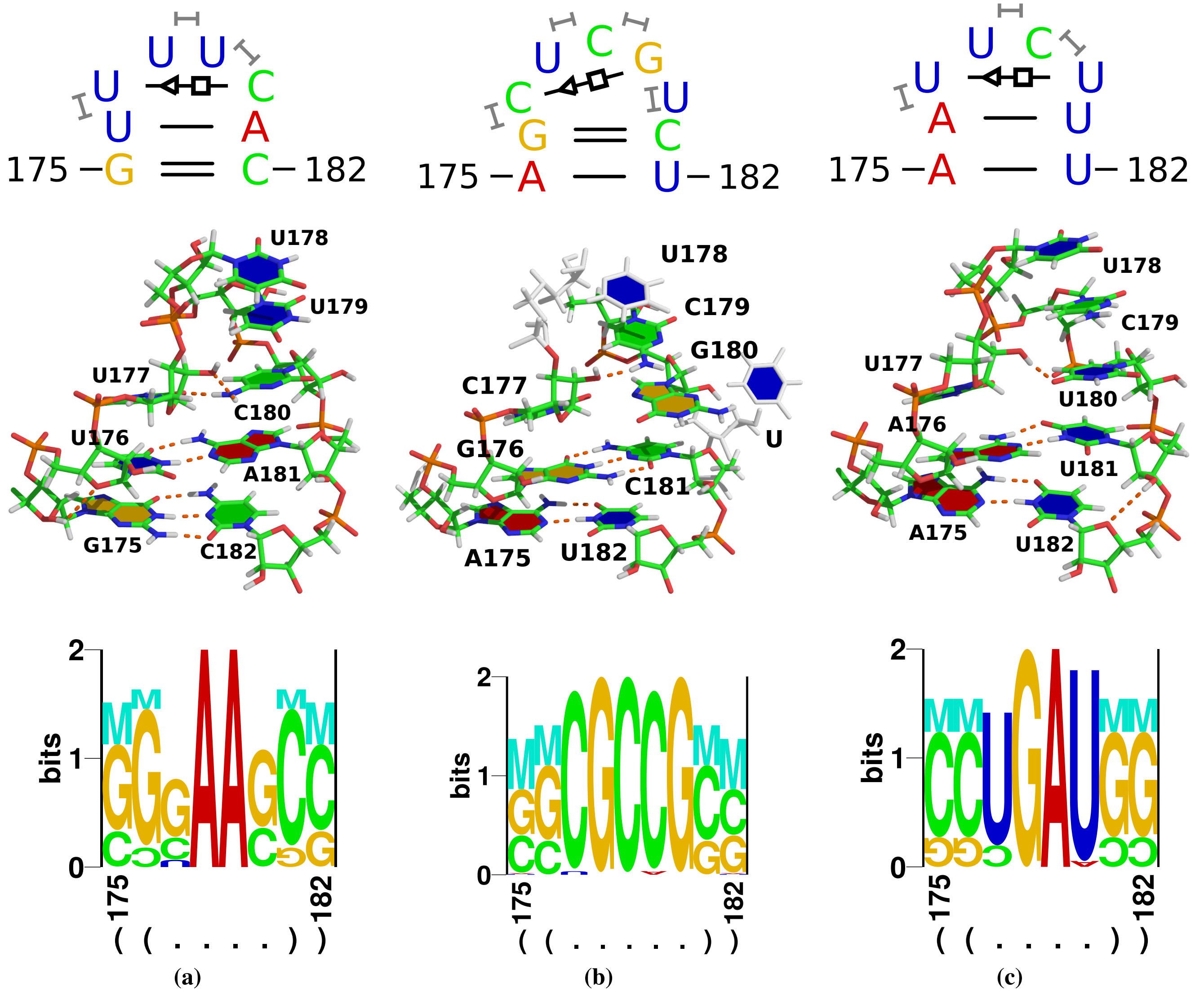

Figure S3. Results of ROSETTA modeling of HP loop. Results for a typical loop of most pospiviroids is shown in (a); a loop typical for CEVd, IrVd, and CBCVd is shown in (b); an exceptional loop of TCDVd is shown in (c). The numbering of the CEVd loop in (b) is arbitrary due the additional loop nucleotide; the numbering of the closing pair, however, fits to the consensus structure (Fig. 1c). Top: Interactions identified by X3DNA-DSSR. Middle: 3D view produced by PYMOL. Bottom: Logos produced by RNA REDESIGN.

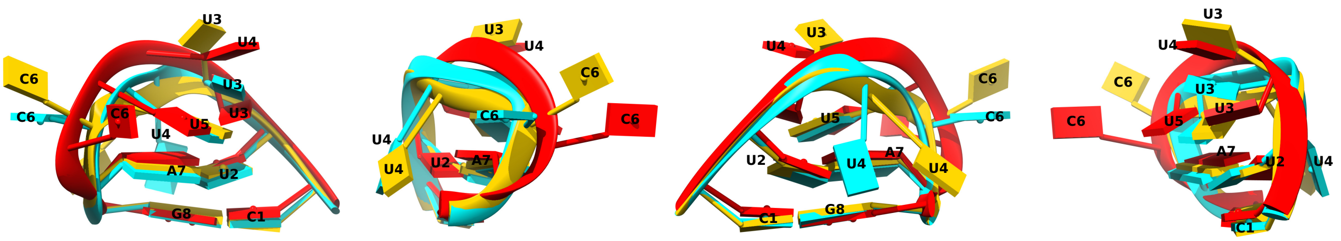

Figure S4. Histone mRNA stem-loop. The overlay on the left is identical to Fig. 7a; each further overlay is turned by 90° along the vertical axis.

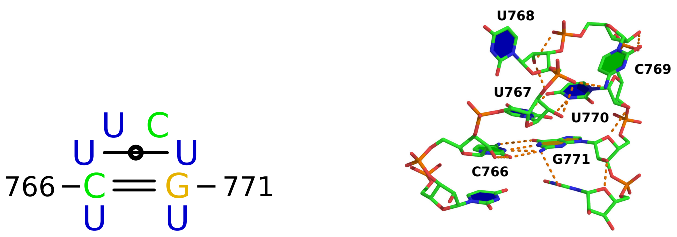

Figure S5. Structure of a hairpin loop from the *Triticum aestivum* 80 S ribosome. The model (PDB entry 4V7E[6]) is based on cryo-electron microscopy with a resolution of < 5 Å. The hairpin loop is identical in sequence to TCDVd (GenBank ID GQ169709), but the closing pair is C:G instead of an A:U in the TCDVd.

Table S2. Structures and infectivity of HP variants.[7]

Mutant[a]	R+	1	2	3
Structure(s)[b]	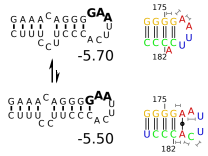	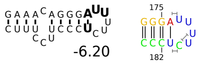	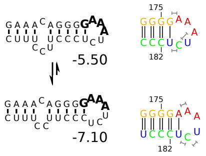	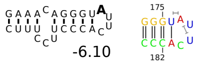
Root[c]	0	2	2	1
Root[d]<br>Leaves[d]	0<br>0	3<br>6	1<br>0	1<br>0

[a]The synthetic variant R+ differs from PSTVd intermediate (WT; AC M88678) by an insertion +G_{176} and two replacements $U_{177}U_{178} \rightarrow$ AA; variants 1–3 are the progeny from *Agrobacterium tumefaciens*-mediated infections of tomato plants with a corresponding plasmid containing a R+ cDNA insertion in (+) orientation.

[b]On the left are given relevant structures and their $\Delta G^{\circ}_{37\,^{\circ}C}$ values (in kcal/mol) as predicted by RNAFOLD;[8] mutations are marked in bold. On the right are give annotations by RNAVIEW of top HP models predicted by ROSETTA.

[c]In 90% of infected plants, viroid progeny was only detected in galls and roots, but not leaves. Numbers give sequence variants present in six cDNA clones from root.

[d]In 10% of infected plants, viroid progeny was detected in galls, roots, and leaves. Numbers give sequence variants present in six cDNA clones from root (upper row) and leaf (lower row), respectively.

Table S3. Predicted structure of IL2 mutants.[9]

Mutant[a]	WT	MT1	MT2	MT3	MT4	MT6
Structure[b]	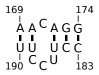	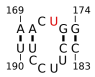	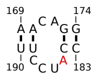	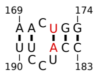	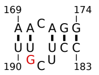	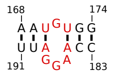
Structure[c]	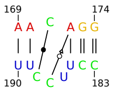	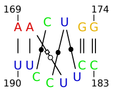	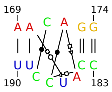	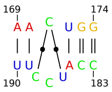	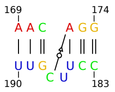	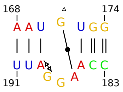
Infectivity[d]	infectious	non-infectious	reverts	non-infectious	non-infectious	non-infectious
Percentage of bound RNA[e]	100	13	16	25	17	16

[a]Wild-type (WT) and mutant (MT1–4, MT6) names; the TR fragments (nt. 145–223) are named R79-wt, R79-mt1–4, and R79-mt6, respectively, in Gozmanova *et al.*[9]

[b]Nucleotides mutated in comparison to WT are in red; these structures were used as input for ROSETTA modelling.

[c]Annotation by RNAVIEW of top models predicted by ROSETTA.

[d]Longer-than-unit-length viroid (based on variant KF440-2, AC X58388) RNA transcripts were assayed for infectivity on tomato plants.[9]

[e]The binding of a Virp1 fragment to TR RNA fragments was determined by EMSA; the fraction of bound WT RNA was set to 100%.[9]

Table S4. Structures and ability to replicate and traffic of synthetic TR mutants.[10]

Mutant[a]	25 (IL1)	26 (IL2)	27 (HP)	
Structure of WT[b]	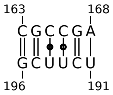	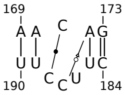	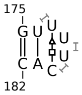	
Structure of mutant[c]	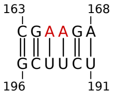	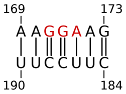	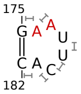	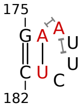
Replication efficiency/%[d]	29	46	53	
Trafficking[e]	0/12	0/12	1/12[f]	

[a]The loops in the secondary structure of PSTVd (see Fig. 1a) were numbered from left to right in Zhong *et al.*;[10] that is, loops 25, 26, and 27 correspond to IL1, IL2, and HP, respectively. The internal loops IL1 and IL2 were closed by canonical WC base pairs; in the HP loop a replacement $U_{176}U \rightarrow AA$ was made.

[b]Annotation by RNAVIEW of top models predicted by ROSETTA for the WT sequence.

[c]Annotation by RNAVIEW of top models predicted by ROSETTA for the mutant sequences. The red letters denote introduced mutations from the WT.

[d]Accumulation level of circular genomic RNA in inoculated protoplasts of *Nicotiana benthamiana* cultured cells expressed as percentage of wild-type PSTVd.

[e]Number of plants showing systemic infection over total number of plants inoculated.

[f]In the one plant that got systemically infected an additional mutation ($A_{181} \rightarrow U$; see structure on the right) occurred that restored the loop-closing basepair.

References

1. Sanchez, R. & Zhou, M. The role of human bromodomains in chromatin biology and gene transcription. *Curr. Opin. Drug Discov. Devel.* **12**, 659–665 (2009).
2. Lin, Y. *et al.* Solution structure of the extraterminal domain of the bromodomain-containing protein BRD4. *Protein Sci.* **17**, 2174–2179 (2008). http://dx.doi.org/10.1110/ps.037580.108.
3. de Castro, E. *et al.* ScanProsite: detection of PROSITE signature matches and ProRule-associated functional and structural residues in proteins. *Nucleic Acids Res.* **34**, W362–5 (2006). http://dx.doi.org/10.1093/nar/gkl124.
4. Maniataki, E., Martínez de Alba, A., Sägesser, R., Tabler, M. & Tsagris, M. Viroid RNA systemic spread may depend on the interaction of a 71-nucleotide bulged hairpin with the host protein VirP1. *RNA* **9**, 346–354 (2003). http://dx.doi.org//10.1261/rna.2162203.
5. Leontis, N. & Westhof, E. Geometric nomenclature and classification of RNA base pairs. *RNA* **7**, 499–512 (2001).
6. Gogala, M. *et al.* Structures of the Sec61 complex engaged in nascent peptide translocation or membrane insertion. *Nature* **506**, 107–110 (2014). http://dx.doi.org/10.1038/nature12950.
7. Hammond, R. *Agrobacterium*-mediated inoculation of PSTVd cDNAs onto tomato reveals the biological effect of apparently lethal mutations. *Virology* **201**, 36–45 (1994). http://dx.doi.org/10.1006/viro.1994.1263.
8. Lorenz, R. *et al.* ViennaRNA Package 2.0. *Algorithms Mol. Biol.* **6**, 26 (2011). http://dx.doi.org/10.1186/1748-7188-6-26.
9. Gozmanova, M., Denti, M., Minkov, I., Tsagris, M. & Tabler, M. Characterization of the RNA motif responsible for the specific interaction of potato spindle tuber viroid RNA (PSTVd) and the tomato protein Virp1. *Nucleic Acids Res.* **31**, 5534–5543 (2003). http://dx.doi.org/10.1093/nar/gkg777.
10. Zhong, X., Archual, A., Amin, A. & Ding, B. A genomic map of viroid RNA motifs critical for replication and systemic trafficking. *Plant Cell* **20**, 35–47 (2008). http://dx.doi.org/10.1105/tpc.107.056606.
